# Localization of Ferromagnetic Target with Three Magnetic Sensors in the Movement Considering Angular Rotation

**DOI:** 10.3390/s17092079

**Published:** 2017-09-11

**Authors:** Xiang Gao, Shenggang Yan, Bin Li

**Affiliations:** 1 School of Marine Science and Technology, Northwestern Polytechnical University, 710072 Xi’an, China; yshgang@nwpu.edu.cn (S.Y.); libin_cme@nwpu.edu.cn (B.L.); 2 Science and Technology on Near-Surface Detection Laboratory, 214035 Wuxi, China

**Keywords:** magnetic detection techniques, ferromagnetic target, the magnetic sensor array, trajectory localization estimation, rotation angle

## Abstract

Magnetic detection techniques have been widely used in many fields, such as virtual reality, surgical robotics systems, and so on. A large number of methods have been developed to obtain the position of a ferromagnetic target. However, the angular rotation of the target relative to the sensor is rarely studied. In this paper, a new method for localization of moving object to determine both the position and rotation angle with three magnetic sensors is proposed. Trajectory localization estimation of three magnetic sensors, which are collinear and noncollinear, were obtained by the simulations, and experimental results demonstrated that the position and rotation angle of ferromagnetic target having roll, pitch or yaw in its movement could be calculated accurately and effectively with three noncollinear vector sensors.

## 1. Introduction

Magnetic positioning technology, with its advantages of all-weather performance, simple equipment, convenient signal processing and so on, is widely applied in the fields of geological exploration, biomedical treatment, wreck removal and localization of unexploded ordinance [1].

Magnetic sensor arrays are commonly used to estimate the location of moving ferromagnetic objects. As early as 1975, the superconducting gradiometer had been used by the United States Naval Research Laboratory to locate the moving magnetic dipole source. Wynn utilized magnetic gradient tensor data to track magnetic dipole, and realized the motion tracking of magnetic dipole with continuous measurement data of static measuring station [2,3]. Subsequently, a large number of methods have been developed to locate the target with magnetic sensor arrays. In 2003, Heath constructed algorithms in MATLAB for the three-dimensional inversion of potential field tensor data using Monte Carlo and Downhill Simplex approaches, while these algorithms have a set target misfit and the final geological models are illustrated in three dimensions [4]. In 2006, Nara showed a simple reconstruction formula for localization of a magnetic dipole whatever the posture of the dipole is. Additionally, he used the developed sensor unit measuring three components of the magnetic field and six components of the spatial gradient tensor at a single place simultaneously to realize the localization [5]. In 2007, Arie formulated the problem as an over-determined nonlinear equation set using a magnetic dipole model for the target and used simulated annealing in order to rapidly find a good approximation to the global optimum of this equation set [6]. In 2009, Wei presented a new mono-component magnetic localization method with a hybrid optimization algorithm, in which a new objective function was constructed to utilize the vertical magnetic field of a vessel [7]. In 2010, Oruc investigated the maxima of the magnitude of magnetic vector components and analytic signals of magnetic gradient tensor resulting from point-dipole and line of dipole sources in determining horizontal locations [8]. In 2011, Tang proposed an algorithm to transform the azimuth estimation problem into the problem of measuring the direction of target magnetic field and its deviation to the azimuth. In order to achieve better real-time detection of underwater magnetic target more efficiently [9], Yu proposed a new method to make use of the array of magnetometers to localize the underwater magnetic target, and it has the virtue of having a simple operation structure and strong real-time detection [10]. In 2014, Wahlstrom indicated that the sensor models could be combined with a standard motion model and a standard nonlinear filter to track metallic objects in a magnetometer network [11]. In 2015, Roger developed a dedicated genetic algorithm to localize the trajectory of ferromagnetic moving objects within a bounded perimeter [12]. In order to solve the problem of geometric parameter transformation of the magnetic gradient mathematical model, Han designed a new mixed algorithm by utilizing the Particle swarm optimization and Newton optimizing method [13]. In 2017, Authors proposed a method of target localization with the alternating magnetic field based on coherent demodulation, but the single alternating magnetic dipole should have no roll and move at a constant speed [14]. However, these methods all focus on the localization of a moving ferromagnetic object which has no roll, pitch or yaw in its movement.

In this work, a method for the moving ferromagnetic target localization with three magnetic sensors is proposed. Using the magnetic field data acquired by three noncollinear magnetic sensors, the relative position and rotation angle between the magnetic object and the sensor array could be obtained rapidly and accurately when the magnetic object has roll, pitch or yaw in its movement.

## 2. Localization Algorithm Description

### 2.1. The Model of Magnetic Dipole

**Figure 1:**
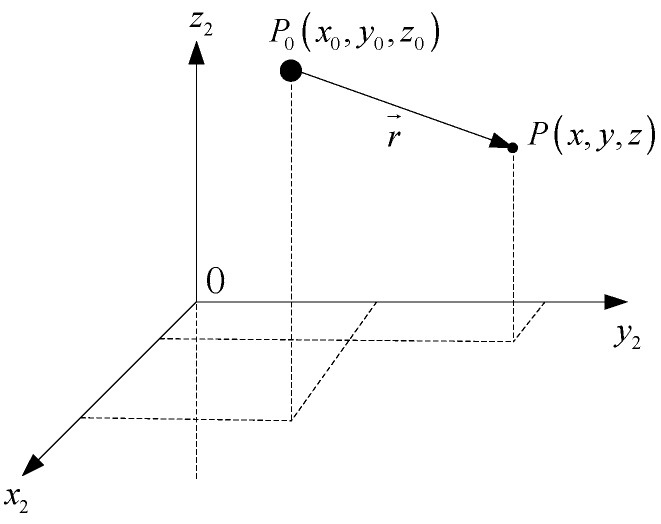
The schematic diagram of a magnetic dipole.

As shown in Figure 1, the magnetic target in the point $P_{0}(x_{0},y_{0},z_{0})$ of the coordinate could be equivalent to a magnetic dipole model labeled as [15]

(1) $$\vec{M_{0}}=M_{x0}\vec{i}+M_{y0}\vec{j}+M_{z0}\vec{k}$$

The magnetic vector potential and magnetic fields at the point P(x,y,z) could be described as

(2) $$\mu (x,y,z)=\frac{\vec{M_{0}}\cdot \vec{r}}{4\pi r^{3}}$$

(3) $$\vec{H}=-grad(\mu )=\frac{1}{4\pi r^{3}}\left[\frac{3}{r^{2}}\left(\vec{M_{0}}\cdot \vec{r}\right)\vec{r}-\vec{M_{0}}\right]$$

where, $r=\sqrt{{\left(x-x_{0}\right)}^{2}+{\left(y-y_{0}\right)}^{2}+{\left(z-z_{0}\right)}^{2}}$.

The magnetic fields acquired by the three-component sensor at the point P(x,y,z) could be calculated according to the following relation:

(4) $$\left[\begin{array}{l} H_{x2}\\ H_{y2}\\ H_{z2} \end{array}\right]=\frac{1}{4\pi r^{5}}\left[\begin{array}{ccc} \left[3{\left(x-x_{0}\right)}^{2}-r^{2}\right] & 3(x-x_{0})(y-y_{0}) & 3(x-x_{0})(z-z_{0})\\ 3(x-x_{0})(y-y_{0}) & 3[{\left(y-y_{0}\right)}^{2}-r^{2}] & 3(y-y_{0})(z-z_{0})\\ 3(x-x_{0})(z-z_{0}) & 3(y-y_{0})(z-z_{0}) & 3[{\left(z-z_{0}\right)}^{2}-r^{2}] \end{array}\right]\left[\begin{array}{l} M_{x0}\\ M_{y0}\\ M_{z0} \end{array}\right]$$

### 2.2. The Rotation Angle Between the Magnetic Dipole and Sensor Array

**Figure 2:**
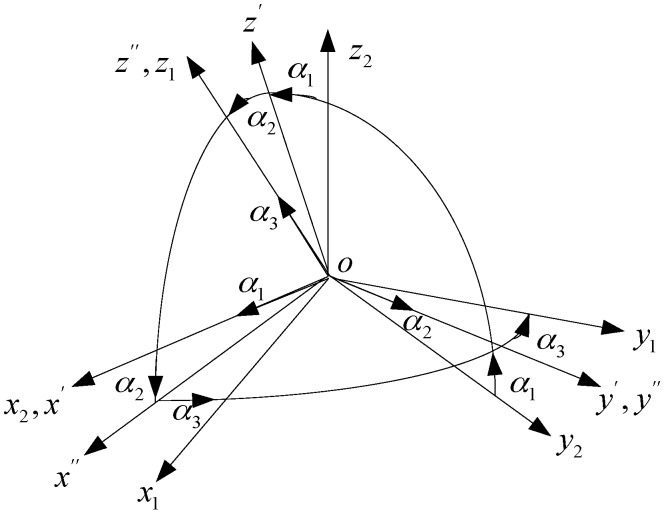
Coordinate rotation of magnetic dipole and magnetic sensor.

There are angle deviations between the three-component magnetic moments of the magnetic dipole and the three axes of the magnetic sensor array. As shown in Figure 2, it is assumed that $\alpha_{1}$ is the first rotation angle around the axis $x^{\prime }$, $\alpha_{2}$ is the second rotation angle around the axis $y^{\prime }$, $\alpha_{3}$ is the third rotation angle around the axis $z^{\prime \prime }$. Then, the coordinate system labeled as $x_{2}y_{2}z_{2}$ is rotated to the coordinate system labeled as $x_{1}y_{1}z_{1}$.

The magnetic field components $H_{x1}$, $H_{y1}$ and $H_{z1}$ are extracted from the sensing signals. As well, $H_{x2}$, $H_{y2}$ and $H_{z2}$ are the magnetic field components that parallels to the coordinate axes $x_{2}$, $y_{2}$ and $z_{2}$, respectively. The relationship between ($H_{x1}$, $H_{y1}$, $H_{z1}$) and ($H_{x2}$, $H_{y2}$, $H_{z2}$) is as follows:

(5) $$\left[\begin{array}{l} H_{x1}\\ H_{y1}\\ H_{z1} \end{array}\right]=C\left[\begin{array}{l} H_{x2}\\ H_{x2}\\ H_{x2} \end{array}\right]$$

here

(6) $$\begin{array}{ll} C & =\left[\begin{array}{ccc} \cos\alpha_{3} & \sin\alpha_{3} & 0\\ -\sin\alpha_{3} & \cos\alpha_{3} & 0\\ 0 & 0 & 1 \end{array}\right]\left[\begin{array}{ccc} \cos\alpha_{2} & 0 & -\sin\alpha_{2}\\ 0 & 1 & 0\\ \sin\alpha_{2} & 0 & \cos\alpha_{2} \end{array}\right]\left[\begin{array}{ccc} 1 & 0 & 0\\ 0 & \cos\alpha_{1} & \sin\alpha_{1}\\ 0 & -\sin\alpha_{1} & \cos\alpha_{1} \end{array}\right]\\ & =\left[\begin{array}{ccc} \cos\alpha_{2}\cos\alpha_{3} & \sin\alpha_{1}\sin\alpha_{2}\cos\alpha_{3}+\cos\alpha_{1}\sin\alpha_{3} & -\cos\alpha_{1}\sin\alpha_{2}\cos\alpha_{3}+\sin\alpha_{1}\sin\alpha_{3}\\ -\cos\alpha_{2}\sin\alpha_{3} & -\sin\alpha_{1}\sin\alpha_{2}\sin\alpha_{3}+\cos\alpha_{1}\cos\alpha_{3} & \cos\alpha_{1}\sin\alpha_{2}\sin\alpha_{3}+\sin\alpha_{1}\cos\alpha_{3}\\ \sin\alpha_{2} & -\sin\alpha_{1}\cos\alpha_{2} & \cos\alpha_{1}\cos\alpha_{2} \end{array}\right] \end{array}$$

is a rotation matrix defined by the rotation parameters.

### 2.3. The Locating Model of Magnetic Field Signal

Localization of mobile magnetic target could be attributed to the solution for a class of nonlinear unconstrained optimization problem as

(7) $$E_{0}=\min\left\{{\left(F_{0}M_{0}-H_{1}\right)}^{T}(F_{0}M_{0}-H_{1})\right\}$$

where $E_{0}$ is the objective function of the nonlinear unconstrained optimization problem.

(8) $$M_{0}={\left(F_{0}{}^{T}F_{0}\right)}^{-1}F_{0}{}^{T}H_{1}$$

is the coefficient matrix of magnetic moment parameters.

(9) $$H_{1}=\left[\begin{array}{l} H_{x1}\\ H_{y1}\\ H_{z1} \end{array}\right]$$

is the magnetic field signal acquired by the three-component magnetic sensor.

(10) $$F_{0}=\frac{1}{4\pi r^{5}}\left[\begin{array}{ccc} \left[3{\left(x-x_{0}\right)}^{2}-r^{2}\right] & 3(x-x_{0})(y-y_{0}) & 3(x-x_{0})(z-z_{0})\\ 3(x-x_{0})(y-y_{0}) & 3[{\left(y-y_{0}\right)}^{2}-r^{2}] & 3(y-y_{0})(z-z_{0})\\ 3(x-x_{0})(z-z_{0}) & 3(y-y_{0})(z-z_{0}) & 3[{\left(z-z_{0}\right)}^{2}-r^{2}] \end{array}\right]$$

is the coefficient matrix of the target positions.

**Figure 3:**
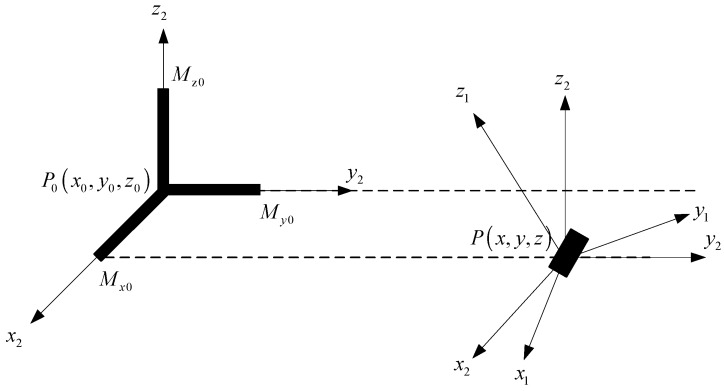
The position relationship between the target and the sensor.

In order to specify the position and rotation angle of the sensor relative to the ferromagnetic target, the coordinate of the position relationship between the target and the sensor is shown in Figure 3.

From a strict mathematical point of view, at least three vector sensors are required since there are nine unknown quantities: the three moment components ($M_{x0}$, $M_{y0}$, $M_{z0}$), the three position coordinates (x, y, z) and the three rotation parameters ($\alpha_{1}$, $\alpha_{2}$, $\alpha_{3}$), and each sensor provides three equations.

## 3. Simulations

**Figure 4:**
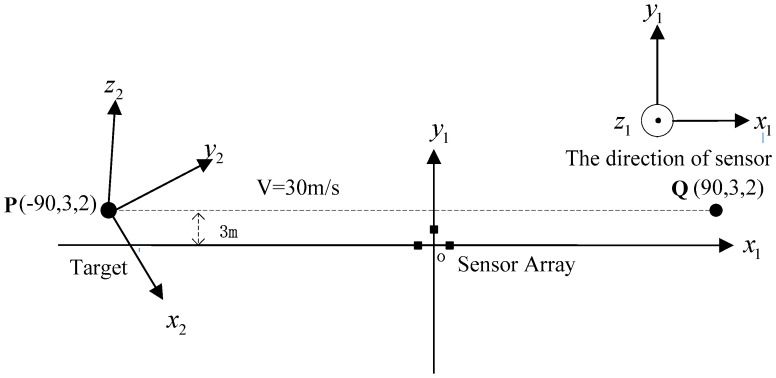
The overhead view of three noncollinear magnetic sensors.

**Figure 5:**
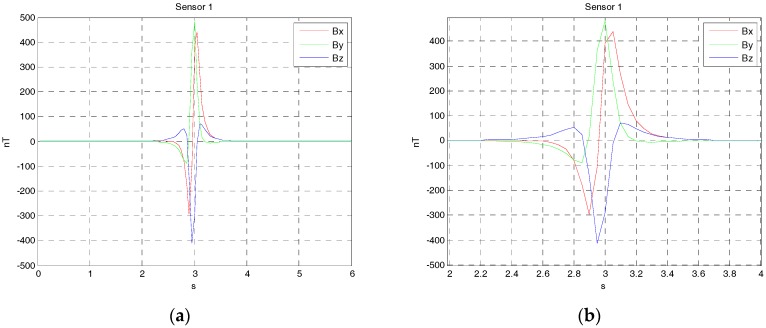
(**a**) The three components alternating magnetic field data acquire by sensor 1 from the time of 0 s to 6 s; (**b**) The three components alternating magnetic field data acquire by sensor 1 from the time of 2 s to 4 s.

**Figure 6:**
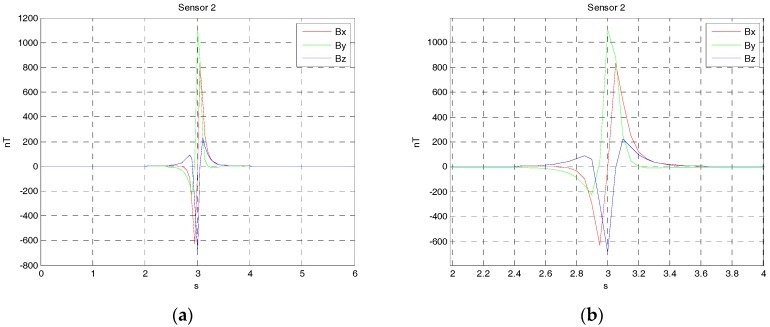
(**a**) The three components alternating magnetic field data acquire by sensor 2 from the time of 0 s to 6 s; (**b**) The three components alternating magnetic field data acquire by sensor 2 from the time of 2 s to 4 s.

**Figure 7:**
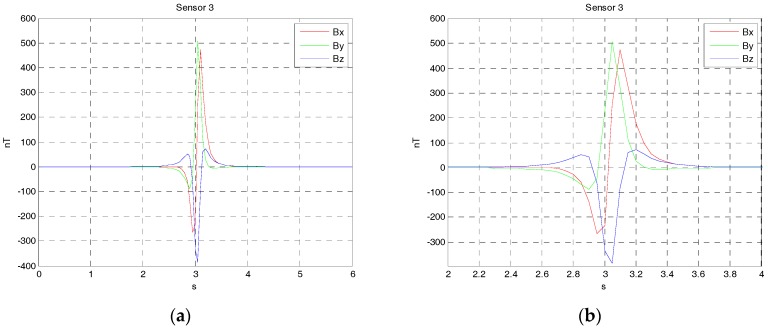
(**a**) The three components alternating magnetic field data acquire by sensor 3 from the time of 0 s to 6 s; (**b**) The three components alternating magnetic field data acquire by sensor 3 from the time of 2 s to 4 s.

Since three vector magnetic sensors are either noncollinear or collinear, the one case is that the magnetic sensors array is arranged at the origin shown in Figure 4. The source of ferromagnetic target is at the point P, which moved from the starting point P(−90,3,2) to the ending point Q(90,3,2) along a straight line at a constant velocity. The velocity is set as 30 m/s. The sensors array fixed to the ground is sampled by a data acquisition module, and the rate was set as 20 Hz. The magnetic moment of the ferromagnetic target was set as $M_{0}=[200\;100\;50]Am^{2}$. The three rotation angles between the magnetic dipole and sensor array is ($60^{\circ }$, $45^{\circ }$, $30^{\circ }$). The sensors 1, 2 and 3 are at points (−1 0 0), (0 1 0) and (1 0 0), respectively. The three vector magnetic field data acquired by the three magnetic sensor arrays is shown in Figure 5, Figure 6 and Figure 7.

**Figure 8:**
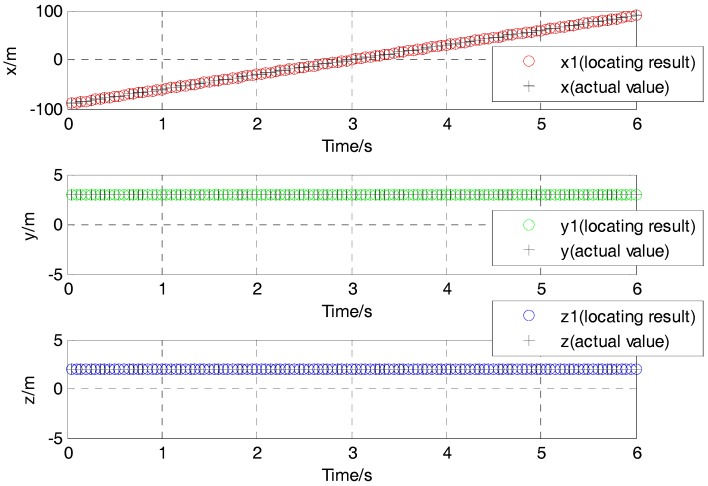
The position of the moving ferromagnetic target considering angular rotation.

**Figure 9:**
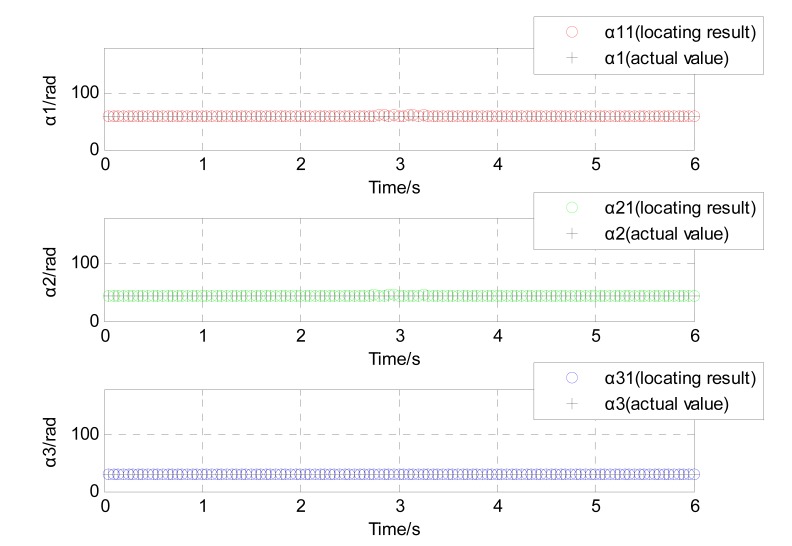
The rotation angle of the moving ferromagnetic target considering angular rotation.

The L-M algorithm was applied to get the position and rotation angle of the moving target with magnetic field data acquired by the sensors array. Using the solution for a class of nonlinear unconstrained optimization problem derived in Equation (7), the results of localization for moving ferromagnetic target which has roll, pitch or yaw in its movement could be shown in Figure 8 and Figure 9.

It could also be stated that the simulation result in the X direction is an oblique line, and the average velocity in the positive X direction is about 30 m/s. The simulation result in the Y direction is a constant value of around 3 m. The simulation result in the Z direction is also a constant value of almost 2 m (see Figure 8). And the rotation angle between the magnetic dipole and sensor array also show a good agreement with the supposed case. The rotation angles around the axis $x^{\prime }$, $y^{\prime }$ and $z^{\prime \prime }$ is $60^{\circ }$, $45^{\circ }$ and $30^{\circ }$, respectively (see Figure 9).

**Figure 10:**
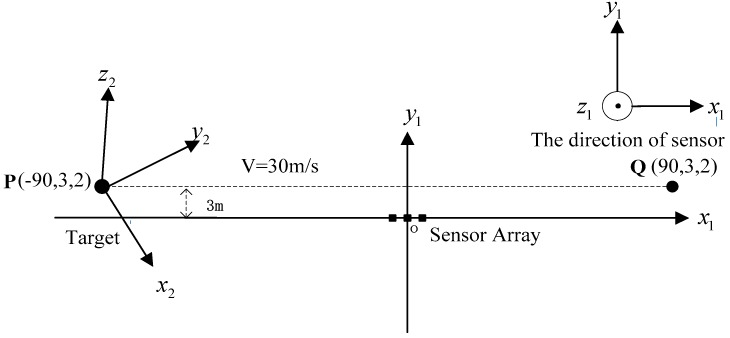
The overhead view of three collinear magnetic sensors.

Since the three magnetic sensors are either noncollinear or collinear, the other case is that the magnetic sensors array is arranged at the origin shown in Figure 10. The sensors 1, 2 and 3 are positioned at (−1 0 0), (0 0 0) and (1 0 0), respectively.

**Figure 11:**
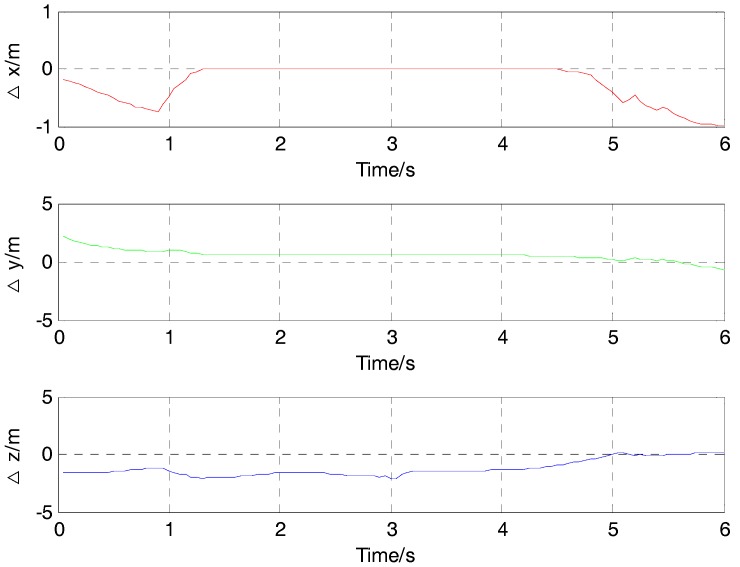
The difference of position between locating results and actual values in three directions.

The locating results of three collinear magnetic sensors in Figure 11 show a disagreement with the supposed case in the simulation. The locating results all have some deviations compared with the actual values. The difference of position between locating results and actual values in the X direction is around 0 m from the time of 1.3 s to 4.5 s, while the value of locating results is different form the actual value in the Y direction and Z direction in the movement. For example, when the time is 1 s, the difference of position between the locating result and actual value is about −0.5 m in the X direction, 0.97 m in the Y direction, and −1.4 m in the Z direction.

**Figure 12:**
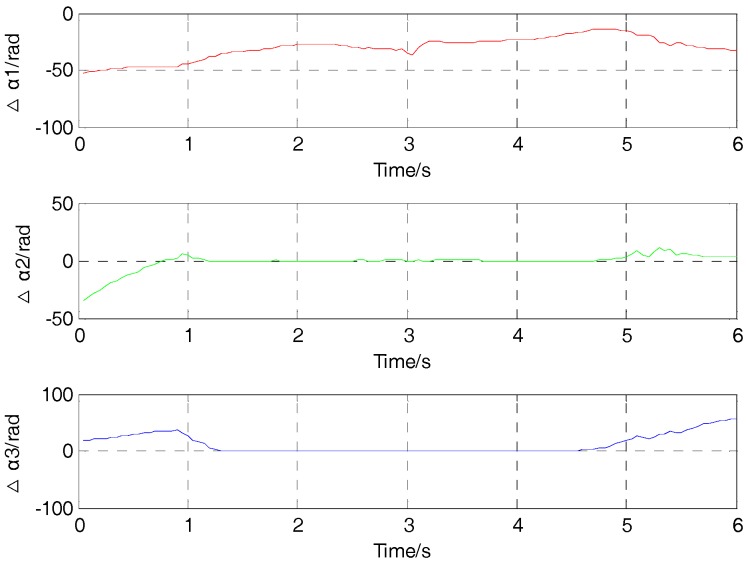
The difference of rotation angle between locating results and actual values in three directions.

As shown in Figure 12, the rotation angle also have some deviations compared with the actual values. The difference of rotation angle between locating results and actual value in the Y direction is around 0° from the time of 1.2 s to 4.7 s. Additionally, the difference of rotation angle between locating results and actual value in the Z direction is around 0° from the time of 1.3 s to 4.6 s, while the value of rotation angle is obviously different form the actual value in the X direction in the movement. For example, when the time is 1 s, the difference of rotation angle between locating results and actual value is about −44.1° in the X direction, 4° in the Y direction, and 25.5° m in the Z direction.

## 4. Experimental Tests

**Figure 13:**
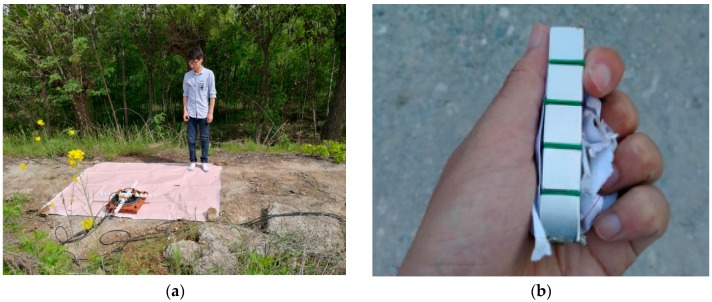
(**a**) The picture of experimental tests; (**b**) The ferromagnetic target taken by a experimenter.

**Figure 14:**
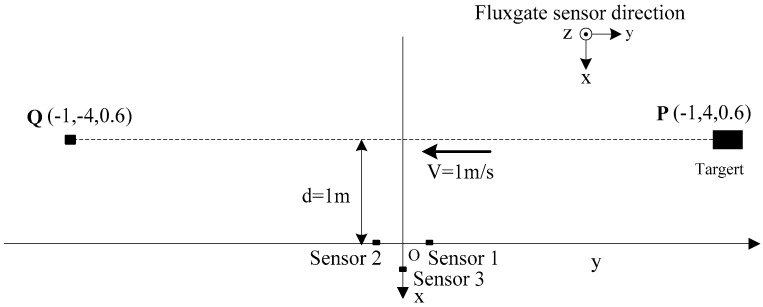
Overhead view of experimental tests.

The experimental site was located in Xi’an, China, where the ambient magnetic disturbance and the magnetic field gradient were very low. The ferromagnetic target was taken by a experimenter with deliberate angular rotation, and the height is about 0.6 m, which moved from P(−1, 4, 0.6) to Q(−1, −4, 0.6) at the velocity of about 1 m/s; the transverse distance is set as 1 m (see Figure 13a). The ferromagnetic target is shown in Figure 13b. The geographic position coordinate between the target and the fluxgate sensors are shown in Figure 14. The sensors 1, 2 and 3 are positioned at (0 0.2 0), (0 −0.2 0) and (0.2 0 0), respectively.

**Figure 15:**
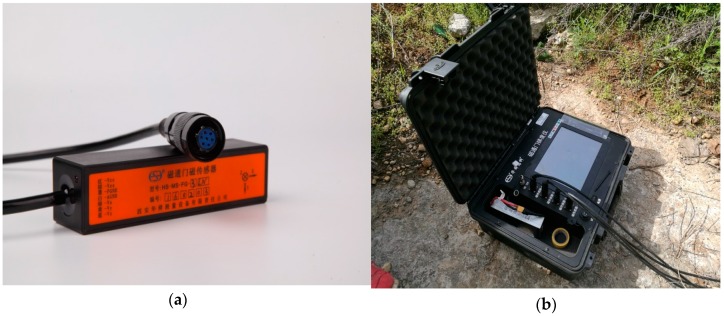
(**a**) Three-component fluxgate sensor of HS-MS-FG-3-LN; (**b**) Data acquisition card of NI 9239.

The magnetic field data of the ferromagnetic target is acquired by three noncollinear fluxgate sensors (HS-MS-FG-3-LN, Xi’an Huashun Measuring Equipment Company, Xi’an, China, see Figure 15a), and is stored by the integrated data acquisition system of fluxgate gradient (HS-MS-GD, Xi’an Huashun Measuring Equipment Company, Xi’an, China, see Figure 15b).

**Figure 16:**
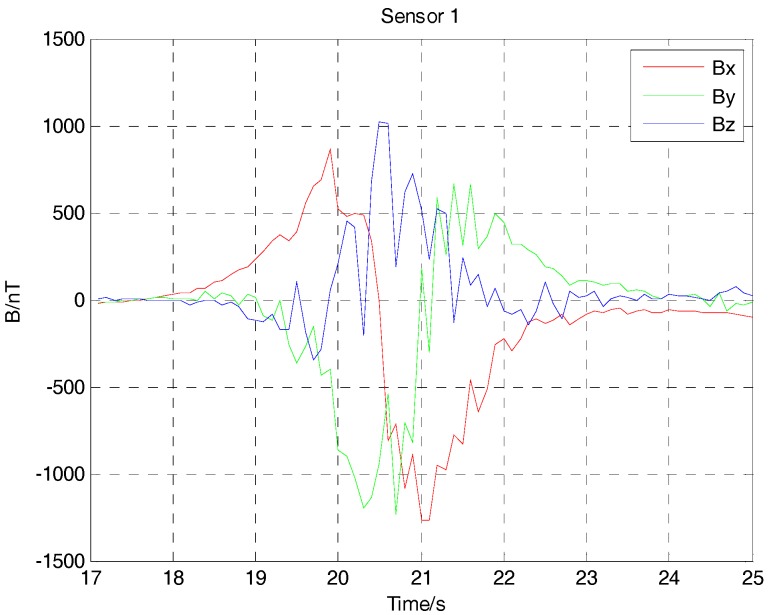
Magnetic field data acquired by three-component fluxgate sensor 1.

**Figure 17:**
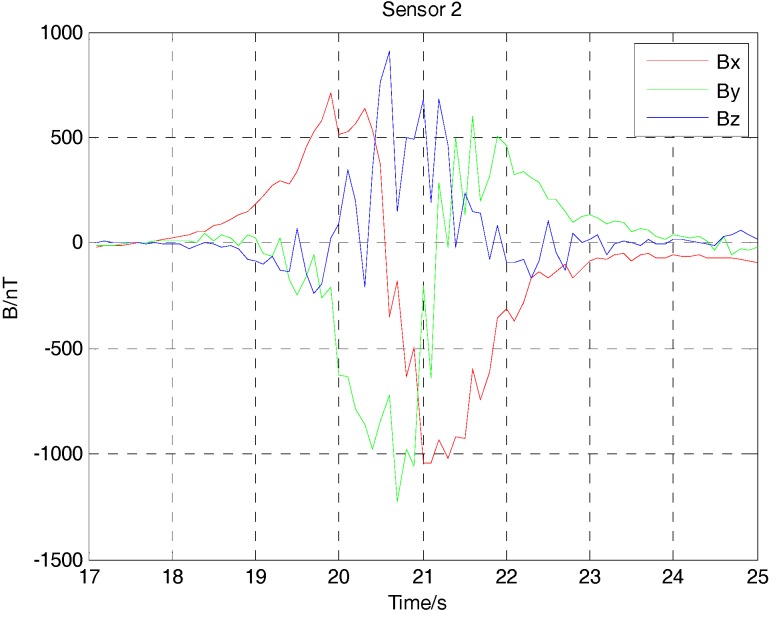
Magnetic field data acquired by three-component fluxgate sensor 2.

**Figure 18:**
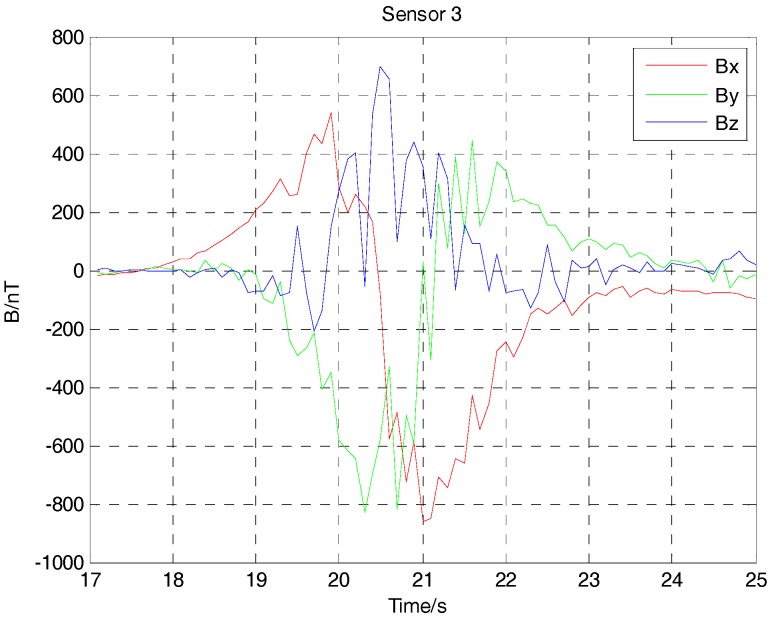
Magnetic field data acquired by three-component fluxgate sensor 3.

Because of the interference of the Earth’s magnetic field, the three-component magnetic field data acquired by three noncollinear fluxgate sensors has a bias. After subtracting the Earth’s magnetic field, the three-component magnetic field data of the ferromagnetic target with deliberate angular rotation in the movement are shown in Figure 16, Figure 17 and Figure 18. Because the ferromagnetic target moved along the *y* axis, the magnetic field data acquired by sensor 1 and sensor 2 should have no differences in the amplitude if we do not consider the time differences. As sensor 3 is farther than sensor 1 and sensor 2 from the target, considering the baseline differences, the magnetic field data acquired by sensor 3 is smaller in amplitude. As the performance of three fluxgate sensors used in the experiment could not be exactly the same, the magnetic fields shown in Figure 16, Figure 17 and Figure 18 have some differences. As the ferromagnetic target was taken by a experimenter with deliberate angular rotation, we could also acquire the rotation angle in the movement.

**Figure 19:**
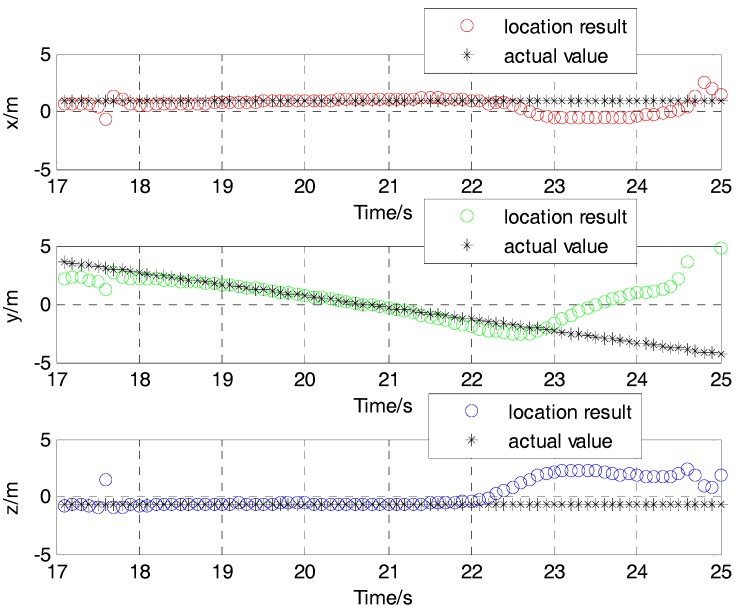
Contrast of location result and actual value in three directions of the experimental tests.

The location results of position for the moving target using the L-M algorithm are shown in Figure 19. The average velocity in the Y direction is about 1.07 m/s from the time of 18 s to 22 s. The corresponding position is about −1 m in the X direction and −0.6 m in the Z direction. These show a good agreement with the actual value from 18 s to 22 s, and a disagreement in the other times. This is the reason that the magnetic field signal gradually increases as the distance of the target and the sensor become close.

**Figure 20:**
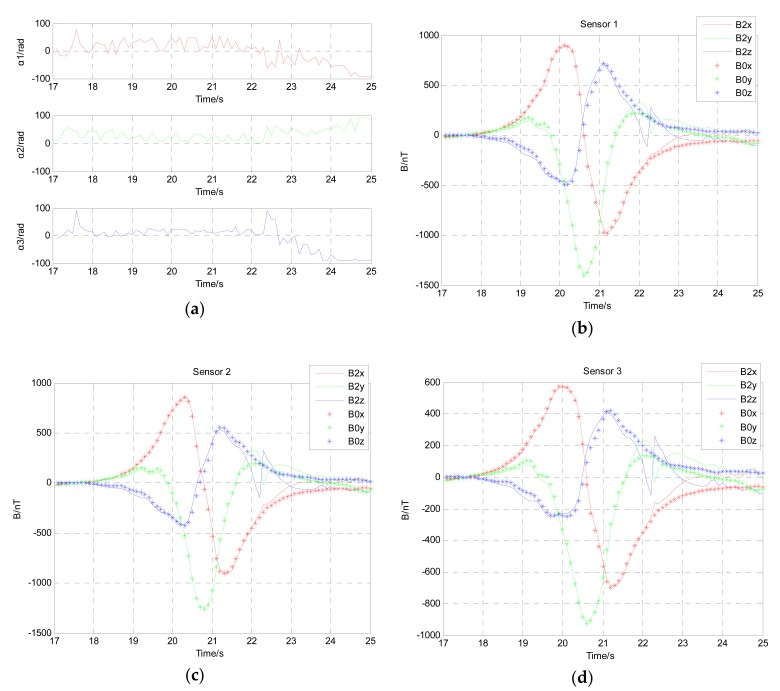
(**a**) The rotation angle between the moving ferromagnetic target and the magnetic sensor; (**b**) Contrast of the magnetic field calibrated by angular data and the actual magnetic field without angular rotation acquired by magnetic sensor 1; (**c**) Contrast of the magnetic field calibrated by angular data and the actual magnetic field without angular rotation acquired by magnetic sensor 2; (**d**) Contrast of the magnetic field calibrated by angular data and the actual magnetic field without angular rotation acquired by magnetic sensor 3.

The location results of rotation angle for the ferromagnetic target are shown in Figure 20a. In order to verify the accuracy of the angular rotation between the three-component magnetic moments of the magnetic dipole and the three axes of the magnetic sensor array, we have used these angular data to calibrate the magnetic field data of three noncollinear fluxgate sensors in three direction. As shown in Figure 20b–d, the three-component magnetic field signed $B_{2x}$, $B_{2y}$ and $B_{2z}$ calibrated by angular data signed $\alpha_{1}$, $\alpha_{2}$ and $\alpha_{3}$ have a good agreement with the magnetic field signed $B_{0x}$, $B_{0y}$ and $B_{0z}$ of the ferromagnetic target without angular rotation. As well, the magnetic field signed $B_{0x}$, $B_{0y}$ and $B_{0z}$ was measured by the magnetic sensor array under the same experimental condition without deliberate angular rotation. The three-component magnetic field signed $B_{2x}$, $B_{2y}$ and $B_{2z}$ calibrated by angular data signed $\alpha_{1}$, $\alpha_{2}$ and $\alpha_{3}$ all have a peak around the time of 22 s in Figure 20b–d. There may be the interference of magnetic noise and clutter in the measurement environment.

## 5. Conclusions

The technology of magnetic detection is widely used in civil and military applications. A method of target localization for a moving objective with three magnetic sensors considering angular rotation was proposed in this paper. The simulations shown that the three noncollinear vector sensors could obtain the position and rotation angle more accurately than that of the three collinear vector sensors. The localization results of ferromagnetic object which has roll, pitch and yaw in its movement also demonstrated the effectiveness of the localization for moving object with three noncollinear magnetic sensors.
